# No Evidence that People Born to Older Parents Show Weaker Preferences for Younger Adult Faces

**DOI:** 10.1007/s40750-025-00263-8

**Published:** 2025-05-20

**Authors:** Jingheng Li, Pengting Lee, Yasaman Rafiee, Benedict C. Jones, Victor K. M. Shiramizu

**Affiliations:** Department of Psychological Sciences & Health, University of Strathclyde, Glasgow, UK

## Abstract

**Purpose:**

People can judge others’ ages from face images somewhat accurately and tend to rateyounger adults’ faces as more attractive than older adults’ faces. However, individual diff erences inthe strength of this preference for younger adult faces have also been reported, whereby peopleborn to older parents (i.e., people whose parents were older when the participant was born) showedweaker preferences for younger adult faces. However, work showing this pattern of results used facestimuli in which cues of age were experimentally manipulated using computer-graphics methods andmany researchers have recently raised concerns about how well fi ndings obtained using such stimuligeneralise to ratings of natural (i.e., unmanipulated) face images.

**Methods:**

In light of the above, we tested whether people born to older parents showed weakerpreferences for younger faces when rating the attractiveness of natural (i.e., unmanipulated) faceimages.

**Results:**

Although our analyses demonstrated that participants generally showed strong preferencesfor younger adult faces, the strength of these preferences was not signifi cantly correlated withparental age at birth.

**Conclusions:**

Thus, our results do not support the proposal that parental age at birth infl uencespreferences for facial cues of age.

## Introduction

People prefer to date, mate with, associate with, hire, and vote for individuals with attractive faces (for reviews see Langlois et al., 2000; Little et al., 2011; Rhodes, 2006). People can assess the ages of individuals somewhat accurately from face photographs, demonstrating that faces contain visual cues of age (e.g., Ebner et al., 2010; Voelkle et al., 2012). Moreover, people tend to rate younger adults’ faces as more attractive than older adults’ faces, suggesting that facial cues of youth are generally preferred (e.g., Foos & Clark, 2011; Perrett et al., 2002).

In a sample of 45 women and 38 men, Perrett et al. (2002) reported that people born to older parents (i.e., people whose parents were older when the participant was born) showed weaker preferences for facial cues of youth. This negative association between parental age at birth and strength of preference for younger adult faces is consistent with other work reporting preferences for other parental characteristics (e.g., hair and eye color) in romantic partners (DeBruine et al., 2017; Little et al., 2003; Marcinkowska & Rantala, 2012) and may be analogous to imprinting-like effects reported in other species (Penn & Potts, 1998; Schielzeth et al., 2008; ten Cate et al., 2006). The function of imprinting-like effects, in which early exposure biases mate preferences in adulthood, are poorly understood, but could include avoiding allocating mating effort to individuals of other species and/or increasing the amount of investment offspring receive from family members (see DeBruine et al., 2008). It is also possible that they are simply a low-cost functionless byproduct of the effects of how visual experience shapes how we process faces and other types of social stimuli (e.g., Buckingham et al., 2006). Understanding of the function of imprinting-like effects on mate preferences is further complicated by the existence of negative imprinting, in which early exposure increases aversions to specific traits or individuals in sexual contexts (DeBruine, 2004, 2005). These negative-imprinting effects could function to increase incest-avoidance (DeBruine, 2004, 2005). Weaker preferences for younger adult faces in individuals born to older parents is also consistent with results of studies demonstrating that visual experience with particular faces can influence facial-attractiveness judgments (Little et al., 2005; Rhodes et al., 2003).

Perrett et al. (2002) reported the negative association between parental age at birth and strength of preference for younger adult faces for attractiveness ratings of face stimuli in which cues of age were experimentally manipulated using computer-graphics methods. However, this methodological approach for manufacturing stimuli for studies of social judgments of faces has recently been criticised for having poor ecological validity (Dong et al., 2023; Jones & Jaeger, 2019; Jones et al., 2023; Lee et al., 2021; Satchell et al., 2023; Scott et al., 2013). Indeed, and consistent with this criticism, several recent studies have reported that findings obtained using stimuli in which facial cues were experimentally manipulated are often qualitatively different to those from studies in which natural (i.e., unmanipulated) face images were rated (Dong et al., 2023; Jones & Jaeger, 2019; Lee et al., 2021; Leger et al., 2023, 2024; Scott et al., 2010). Indeed, several studies have reported that effects obtained using manipulated stimuli are not evident when natural face images are employed (Dong et al., 2023; Jones & Jaeger, 2019; Lee et al., 2021; Leger et al., 2023, 2024; Scott et al., 2010). For example, the large effects of sexual dimorphism on perceptions of attractiveness, trustworthiness, and dominance that have been widely reported in studies using stimuli that were experimentally manipulated are either non-significant or significant but much smaller in size when natural (i.e., unmanipulated) face images are used (Dong et al., 2023; Jones & Jaeger, 2019; Leger et al., 2023; Scott et al., 2010). This difference in the pattern of results obtained for these two types of stimuli has also been observed in studies of the effects of facial symmetry on social judgments (Lee et al., 2021). In light of this potential criticism of the type of stimuli used by Perrett et al. (2002), here we tested whether people born to older parents showed weaker preferences for younger faces when rating the attractiveness of natural (i.e., unmanipulated) face images.

Whether findings for attractiveness judgments of faces translate into actual partner choices is unclear and somewhat controversial (see, e.g., DeBruine et al., 2006; Wincenciak et al., 2015). Indeed, Eastwick et al. (2025) recently reported that the large sex difference in stated preferences for youth in potential partners (men show stronger preferences for youth than women do) was not evident in partnership decisions following blind dates. In light of this issue, we tested whether people born to older parents also tended to report having older romantic partners.

## Methods

All procedures used were approved by the Department of Psychological Sciences and Health (University of Strathclyde) Ethics Commitee, all work was undertaken in accordance with the Declaration of Helsinki, and all participants provided informed consent.

One hundred and fifty heterosexual men (mean age = 33.87 years, SD = 6.34 years), all of whom reported currently being in a romantic relationship (mean relationship duration = 8.45 years, SD = 5.66 years), and 150 heterosexual women (mean age = 34.48 years, SD = 6.27 years), all of whom also reported currently being in a romantic relationship (mean relationship duration = 10.97 years, SD = 6.74 years), took part in the study. Participants were recruited via Prolific and the study was carried out using Qualtrics. This sample size exceeds Simonsohn’s (2015) recommended sample size for replication studies. Simonsohn (2015) recommended replications test 2.5 times the sample size used in the original study (Perrett et al., 2002 tested 35 men and 48 women).

Male participants rated the attractiveness of 50 female faces (mean age = 37.5 years, SD = 13.25 years, range = 19 to 55 years) and female participants rated the attractiveness of 50 male faces (mean age = 35.06 years, SD = 12.46 years, range = 20 to 54 years) using a 1 (not at all) to 7 (very) scale. Trial order was fully randomised, and the attractiveness-rating task was self-paced. The face images used in this attractiveness-rating task had been randomly selected from images of individuals between 19 and 55 years of age in an open-access face-image database (Ebner et al., 2010). Images were full colour and individuals depicted in the images were posed front on to the camera, with neutral expressions, and direct gaze. Clothing was standardised across individuals. Inter-rater agreement was high for attractiveness ratings of both male (Cronbach’s alpha = 0.994) and female (Cronbach’s alpha = 0.996) faces. The mean attractiveness rating for male faces was 2.82 (SD = 1.50) and the mean attractiveness rating for female faces was 2.79 (SD = 1.52).

Immediately after completing the attractiveness-rating task, participants completed a questionnaire in which they reported their romantic partner’s age, mother’s age, and father’s age. Descriptive statistics for these variables are given in Table 1. Participants reported living with their mother until they were, on average, 21.28 years of age (SD = 5.83 years) and reported living with their father until they were, on average, 18.68 years of age (SD = 8.46 years).

**Table 1:** Descriptive statistics (means and SDs) for participant age, romantic partner age, mother’s age, and father’s age

	Male participants	Female participants
Mean romantic partner age (SD)	32.61 years (6.65 years)	36.60 years (7.43 years)
Mean mother’s age (SD)	62.77 years (7.64 years)	61.83 years (7.50 years)
Mean father’s age (SD)	64.57 years (7.61 years)	64.94 years (7.79 years)

For each participant, we calculated their mother’s age when the participant was born (M = 28.12 years, SD = 5.56 years) and their father’s age when the participant was born (M = 30.58 years, SD = 6.21 years). Because these two scores were highly correlated (*r* =.70, *p* <.001), we calculated the average of these two scores for each participant to create a new variable (parental age at birth) that we used in our analyses (M = 29.35 years, SD = 5.43 years). This procedure is identical to that used by Perrett et al. (2002) to create the parental age at the participant’s birth variable in their analyses. Parental age at birth and participant age were weakly but significantly correlated (*r* = −.24, *p* <.001). Table 2 shows the intercorrelations among all continuous variables (95% CIs shown in parentheses).

**Table 2:** Intercorrelations among all continuous variables (95% CIs shown in parentheses)

Variable	1	2	3	4	5
1. Participant age	1				
2. Partner age	0.81*** (0.77, 0.85)	1			
3. Maternal age at birth	− 0.19** (-0.30, − 0.08)	− 0.13* (-0.24, − 0.02)	1		
4. Paternal age at birth	− 0.25*** (-0.35, − 0.14)	− 0.16** (-0.27, − 0.05)	0.70*** (0.64, 0.76)	1	
5. Parental age at birth	− 0.24*** (-0.34, − 0.13)	− 0.16** (-0.27, − 0.05)	0.91*** (0.89, 0.93)	0.93*** (0.91, 0.94)	1

Note. * *p* <.05, ** *p* <.01, *** *p* <.001

## Results

All analyses were carried out using R (R Core Team, 2022), with the packages kableExtra 1.3.4 (Zhu, 2021), lme4 (Bates et al., 2015), lmerTest 3.1-3 (Kuznetsova et al., 2017), jtools 2.2.3 (Long, 2022), psych 2.2.5 (Revelle, 2023), interactions 1.2.0 (Long, 2024), rstatix 0.7.2 (Kassambara, 2023), parameters 0.24.2 (Lüdecke et al., 2020), performance 0.13.0 (Lüdecke et al., 2021), apaTables 2.0.8 (Stanley, 2021), and tidyverse 1.3.1 (Wickham et al., 2019). All data, full outputs, and analysis code are publicly available on the Open Science Framework (https://osf.io/bmfhy/).

### Does Parental Age at the Participant’s Birth Predict Preferences for Facial Cues of Age?

To address this research question, attractiveness ratings were analysed using a linear mixed effects model. The model included participant sex (effect coded so that + 0.5 corresponded to male and − 0.5 corresponded to female), parental age at the participant’s birth, participant age, and stimulus age as predictors, along with the two-way interactions between participant sex and parental age at the participant’s birth, between participant sex and participant age, between participant sex and stimulus age, between participant age and stimulus age, and between parental age at the participant’s birth and stimulus age, and the three-way interactions among participant sex, stimulus age, and parental age at the participant’s birth, and among participant sex, stimulus age, and participant age. The model also included by-subject random intercepts, by-stimuli random intercepts, by-subject random slopes for stimulus age, and by-stimuli random slopes for parental age at the participant’s birth. All continuous predictors were converted to z scores prior to analyses. Although attractiveness ratings were generally somewhat low, skewness (0.58, SE = 0.02) was comfortably within the acceptable range (Tabachnick & Fidell, 2013).

This analysis showed a significant negative effect of stimulus age, indicating that participants generally preferred younger faces. There was also a significant positive effect of participant age, indicating that older participants generally gave higher attractiveness ratings. No other effects or interactions were significant. The full results of this analysis are summarised in Table 3. Importantly, this analysis showed no evidence that participants born to older parents showed significantly weaker preferences for younger faces. Pseudo R squared (fixed effects) for this model was 0.33. Removing participant age from the model showed the same pattern of results as our initial model (see https://osf.io/bmfhy/ for full results of this analysis). Although the interaction between parental age at the participant’s birth and stimulus age was not significant in our initial analysis (*p* =.078), we decomposed it using simple slope analysis. This simple slopes analysis showed that age at photo shoot was significantly and negatively associated with attractiveness ratings in participants born to parents one SD younger than the mean (b = − 0.81, SE = 0.06, t = − 13.55, *p* <.001), at the mean (b = − 0.84, SE = 0.06, t = − 14.74, *p* <.001) and one SD above the mean (b = − 0.88, SE = 0.06, t = − 14.41, *p* <.001). Note that the strength of this association increased as parental age at the participant’s birth increased. Importantly, these analyses showed that participants born to older parents tended to show stronger preferences for younger adult faces, rather than the weaker preferences for younger adult faces reported by Perrett et al. (2002).

**Table 3:** Results of our linear mixed effects model testing whether parental age at birth predicted preferences for facial cues of age

	Estimate	SE	t	df	*p*
(Intercept)	2.814	0.068	41.454	218.372	< 0.001
participant sex	0.151	0.136	1.111	218.372	0.268
parental age at the participant’s birth	-0.008	0.043	-0.176	302.903	0.860
stimulus age	-0.844	0.057	-14.744	121.464	< 0.001
participant age	0.130	0.043	3.060	306.644	0.002
participant sex x parental age at the participant’s birth	-0.115	0.086	-1.347	302.903	0.179
participant sex x stimulus age	0.014	0.114	0.123	121.464	0.902
parental age at the participant’s birth x stimulus age	-0.035	0.020	-1.766	298.186	0.078
participant sex x participant age	0.038	0.085	0.443	306.644	0.658
stimulus age x participant age	-0.015	0.020	-0.765	305.673	0.445
participant sex x parental age at the participant’s birth x stimulus age	0.045	0.040	1.118	298.186	0.265
participant sex x participant age x stimulus age	-0.026	0.040	-0.634	305.673	0.527

We repeated the analysis described above, replacing parental age at the participant’s birth with maternal age at the participant’s birth and repeated it again replacing parental age at the participant’s birth with paternal age at the participant’s birth. We also repeated all of these analyses of attractiveness ratings, replacing stimulus age with the mean perceived (i.e., rated) age for each face. Perceived age data for these analyses were taken from Ebner et al. (2010) open-access data. These alternative analyses showed the same pattern of results as our initial analysis and are reported in full at https://osf.io/bmfhy/. Perceived age and actual age of the individuals photographed was highly and significantly correlated (*r* =.96, *p* <.001).

### Does Parental Age at Birth Predict Actual Partner Age?

To address this research question, partner age data were analysed using linear regression. The model included participant sex (effect coded so that + 0.5 corresponded to male and − 0.5 corresponded to female), parental age at birth, and participant age, as predictors, along with the two-way interactions between participant sex and parental age at birth and between participant sex and participant age. There was a significant positive effect of participant age, indicating that older participants tended to have older partners. There was also a significant effect of participant sex, indicating that female participants’ partners tended to be older than male participants’ partners. No other effects or interactions were significant. The full results of this analysis are summarised in Table 4. Importantly, this analysis showed no evidence that participants born to older parents had older partners. Pseudo R squared (fixed effects) for this model was 0.32. Removing participant age from the model showed the same pattern of results as our initial model (see https://osf.io/bmfhy/ for full results of this analysis).

**Table 4:** Results of our regression analysis testing whether parental age at birth predicted actual partner age

	Estimate	SE	t	*p*
(Intercept)	34.744	0.229	151.553	< 0.001
participant age	5.915	0.235	25.189	< 0.001
participant sex	-3.478	0.459	-7.585	< 0.001
parental age at the participant’s birth	0.376	0.237	1.584	0.114
participant sex x participant age	-0.166	0.470	-0.353	0.724
participant sex x parental age at the participant’s birth	0.324	0.475	0.683	0.495

We repeated the analysis described above, replacing parental age at the participant’s birth with maternal age at the participant’s birth and repeated it again replacing parental age at the participant’s birth with paternal age at the participant’s birth. These alternative analyses showed the same pattern of results as our initial analysis and are reported in full at https://osf.io/bmfhy/.

## Discussion

Consistent with findings from many previous studies (e.g., Foos & Clark, 2011; Perrett et al., 2002), we found that participants rated younger adult faces to be more attractive than older adult faces. However, we found no evidence that the strength of this preference for younger adult faces was significantly correlated with parental age at participant’s birth. This null result for parental age at birth contrasts with Perrett et al. (2002), who found that people born to older parents (i.e., people whose parents were older when the participant was born) showed weaker preferences for younger adult faces. Indeed, a simple slopes analysis we conducted to decompose the non-significant interaction between parental age at the participant’s birth and stimulus age (*p* =.078) indicated that participants in our study who were born to older parents tended to show *stronger* preferences for younger adult faces, rather than the *weaker* preferences for younger adult faces previously reported by Perrett et al. (2002).

Why do our findings for preferences for facial cues of age and parental age at birth differ from those reported by Perrett et al. (2002)? One possibility is that the differences in results between these two studies reflect differences in the type of face stimuli employed. Whereas Perrett et al. (2002) experimentally manipulated visual cues of age in prototype (i.e., synthetic) faces using computer-graphics methods, participants in our study rated the attractiveness of natural (i.e., unmanipulated) faces. This difference is potentially noteworthy, because many researchers have recently raised concerns about the ecological validity of using stimuli in which facial characteristics were experimentally manipulated to investigate social judgments of faces (Dong et al., 2023; Jones & Jaeger, 2019; Jones et al., 2023; Lee et al., 2021; Satchell et al., 2023; Scott et al., 2013). For example, several studies have reported that effects obtained using manipulated stimuli are not evident when natural face images are employed (Dong et al., 2023; Jones & Jaeger, 2019; Lee et al., 2021; Leger et al., 2023, 2024; Scott et al., 2010).

Although we saw only weak evidence that individuals born to older parents show stronger preferences for younger individuals, it is possible that such an effect could be part of a suite of behaviours that function to reduce incest. Negative-imprinting effects have previously been reported in which early exposure is associated with increased aversions in adulthood to familial traits or specific individuals in sexual contexts (DeBruine, 2004, 2005). If people born to older parents do show stronger preferences for younger individuals, it could be a further example of negative imprinting. The factors that influence how individuals resolve the trade-off between the benefits of positive- and negative-imprinting are poorly understood but are a potentially important topic for future research. Considering possible effects of the relationship context for which potential partners are assessed (short- versus long-term relationships) may provide insight into this issue (see DeBruine, 2005 for further discussion). Although we randomly selected images of individuals between 19 and 55 years of age from Ebner et al’s (2010) open-access face-image database to use as stimuIi, Ebner et al’s (2010) image set does not contain images between 32 and 38 years of age. Future studies on this issue may want to ensure stimuli have a fuller representation of ages. The generalizability of imprinting-related effects on attractiveness judgments might also be investigated by considering the role of cultural factors, stimulus and participant ethnicity, and participant sexual orientation.

Consistent with findings from many previous studies (for review see McKenzie, 2021), we found that men reported having younger romantic partners than did women. However, we found no evidence that reported partner age was significantly correlated with parental age at participant’s birth. Thus, we found no evidence that the pattern of results reported by Perrett et al. (2002), whereby people born to older parents (i.e., people whose parents were older when the participant was born) showed weaker preferences for younger adult faces, translates into actual partner choice.

Here we attempted to replicate Perrett et al’s (2002) finding that people born to older parents showed weaker preferences for younger adult faces. Whereas Perrett et al. (2002) used synthetic face stimuli (i.e., prototypes) in which visual cues of age were experimentally manipulated using computer-graphics techniques, participants in our study rated the attractiveness of natural (i.e., unmanipulated) face images. By contrast with Perrett et al’s (2002) results, we found no evidence that parental age at birth was significantly correlated with participants’ preferences for younger adult faces. This pattern of null results was observed both when the actual age of the individuals depicted in the photographs was used as a predictor and when the rated (i.e., perceived) age of the individuals depicted in the photographs was used as a predictor. Thus, our results do not support the proposal that an imprinting-like effect causes parental age at birth to predict preferences for facial cues of age.

## Data Availability

All data, full outputs, and analysis code are publicly available on the Open Science Framework (https://osf.io/bmfhy/).
